# Guanine Crystal Formation at Physiological pH

**DOI:** 10.1021/acs.cgd.5c00205

**Published:** 2025-05-30

**Authors:** Bidisha Tah Roy, Lukas Jorin Hasselt, Ross Young, Zijiang Yang, Jeanine Williams, Johanna M. Galloway, Alex Heyam, Yi-Yeoun Kim, Fiona C. Meldrum

**Affiliations:** School of Chemistry, 4468 University of Leeds, Woodhouse Lane, Leeds LS2 9JT, United Kingdom

## Abstract

Guanine crystals are the principal component of many biocrystals with optical functions. Typically exhibiting unique morphologies and being metastable β anhydrous guanine (β-AG) rather than the thermodynamically stable α anhydrous polymorph (α-AG), many questions remain regarding the mechanisms by which organisms control their formation. However, efforts to elucidate these using bio-inspired approaches have been limited by the very low solubility of guanine in aqueous solutions at physiological pH. Here, we demonstrate an enzymatic approach based on the purine metabolism process that yields significant quantities of guanine crystals in aqueous solution at neutral pH. Significantly, this mirrors processes believed to generate guanine crystals in vivo. The enzyme purine nucleoside phosphorylase (PNP) is used to continuously convert guanosine to guanine and generate supersaturation, and pure β-AG or α-AG can be produced by changing the reagent concentrations or introducing stirring. We also show that the rate of change of supersaturation is crucial in determining the polymorph, demonstrating that organisms can generate β-AG crystals by simply controlling the crystallization conditions. This work bridges the gap between in vitro and biological crystallization and provides a facile means of studying the crystallization of biological molecules and ultimately generating functional materials using sustainable processes.

## Graphical abstract

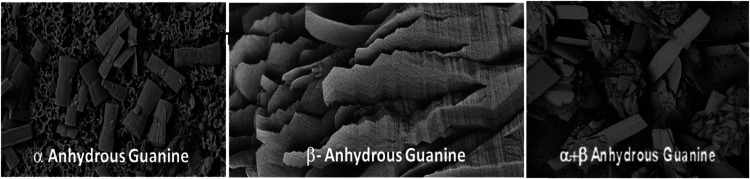

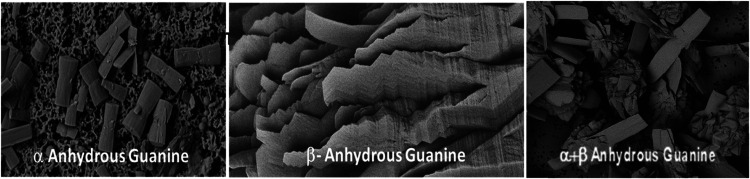

## Introduction

Identification of the strategies by which organisms control the formation of biominerals and their translation to the laboratory is highly attractive, where it promises the ability to generate functional materials using sustainable processes. To date, most work has focused on the abundant inorganic compounds calcium carbonate^1,2^ and calcium phosphate,^3,4^ and has delivered many bioinspired approaches for generating crystals with remarkable morphologies, hierarchical structures, specified polymorphs and superior mechanical properties. By comparison, biocrystals comprising organic molecules have received little attention, even though they also exhibit unique properties that are valuable in synthetic functional materials.^5^ Extensive recent studies have shown that guanine crystals are the principal building blocks of many biocrystals with optical functions, where arrays of these high refractive index crystals can create structural colors and behave as mirrors and broadband or narrowband reflectors in structures such as fish scales,^6,7^ scallop eyes^8^ and chameleon skin.^9^ In common with inorganic biominerals, the guanine crystals form under strict biological control and exhibit sizes, morphologies, and structures that are quite distinct from their synthetic counterparts. They are almost exclusively metastable β anhydrous guanine (β-AG) rather than the stable α anhydrous guanine (α-AG) polymorph typically formed synthetically. These β-AG crystals also commonly take the form of platelets, which contrasts with the prismatic morphology predicted by models.^10,11^

Despite the importance of guanine crystals in biology, many questions remain about the mechanisms that organisms use to control their formation. The development of bioinspired strategies to control guanine crystallization under ambient conditions has also proven extremely challenging due to the very low solubility of guanine in aqueous solutions at neutral pH (15–25 μM).^12^ This contrasts with calcium carbonate and calcium phosphate, which are readily precipitated from aqueous solutions at physiological pH. Most of our knowledge about guanine crystallization has therefore come from studies that use (i) aqueous solutions at high or low pH,^13–15^ or (ii) mixed water/organic solvents such as dimethyl sulfoxide (DMSO)^16^ or formamide^17^ in which guanine is much more soluble.

Here, we introduce a novel strategy that overcomes this problem and delivers a superior yield of guanine crystals in aqueous solution at neutral pH. Importantly, the polymorph of the guanine crystals can be selected by simply varying the solution concentrations or stirring the reaction solution. Our approach is based on the recognition that enzymes are often used by organisms to continuously generate mineral precursors and regulate supersaturation. For example, carbonic anhydrase^18^ and urease^19^ have been implicated in calcium carbonate precipitation in some organisms, while alkaline phosphatase can facilitate calcium phosphate crystallization through the release of Ca^2+^ and PO_4_^3–^ ions.^20,21^ Our process exploits guanosine as a precursor—which is over 100 times more soluble than guanine at neutral pH in phosphate buffer^12^—and uses the enzyme purine nucleoside phosphorylase (PNP) to convert it to guanine and the byproduct ribose-1-phosphate (Scheme 1). This mimics the purine metabolism process that is used to synthesize many nucleotides in biology,^22,23^ where PNP breaks the glycosidic bond of ribose or deoxyribose nucleosides in the presence of inorganic phosphate.^24^ Notably, proteomic analysis of the formation of guanine-based crystals in zebrafish has recently shown an upregulation of the entire guanine metabolic network, consistent with the conversion of guanosine and deoxyguanosine into guanine.^25^ This approach opens the door to the development of strategies for generating guanine crystals resembling their biogenic counterparts in the laboratory.

**Scheme 1:**
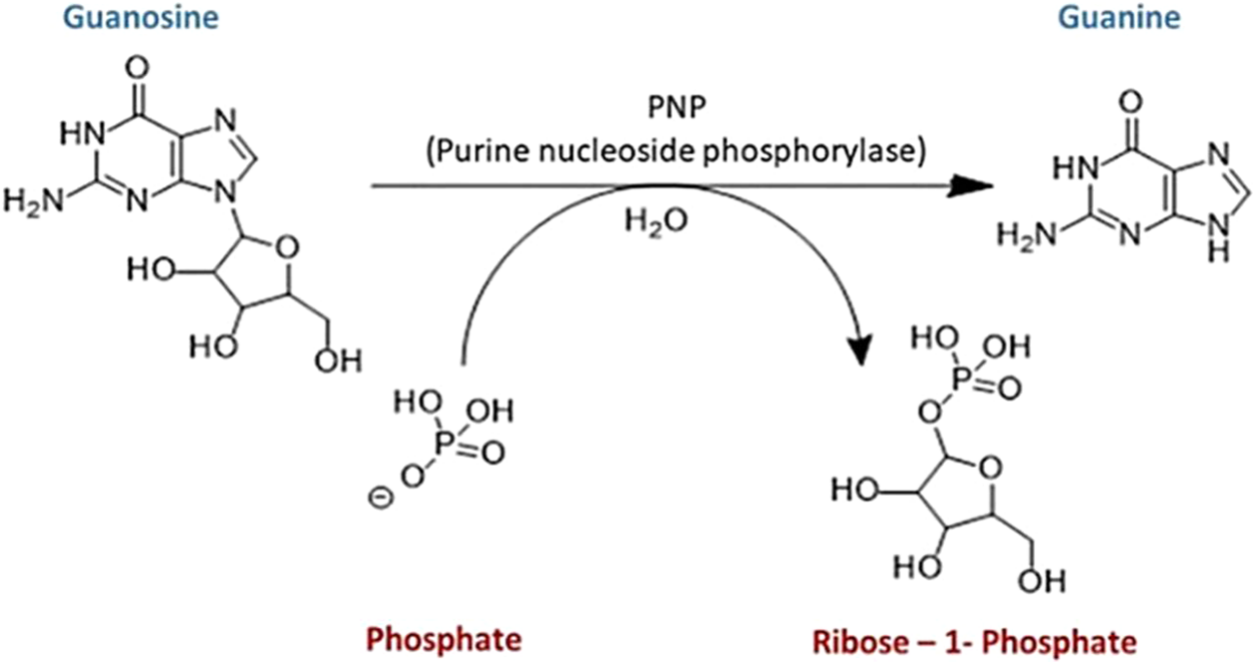
Conversion of Guanosine to Guanine Catalyzed by an Enzyme, Purine Nucleoside Phosphorylase (PNP)

## Results

### Enzymatic Guanine Crystallization

In our standard reaction, 1 unit of PNP enzyme was added to a slightly supersaturated solution of guanosine in a 50 mM phosphate buffer at pH 7.2, and the pH remained constant throughout. The concentration of guanosine under these conditions was 1.6 mM, as measured using high performance liquid chromatography (HPLC), which compares with 13 μM for guanine under comparable conditions.^12,26^ Guanine crystals continuously precipitated in the solution over 2 days until the guanosine was completely consumed. Spherulitic particles that were 5–10 μm in diameter and comprised numerous smaller zigzag-shaped crystals formed after 2 days (Figure 1a,1b). The spherical particles likely originate from spherulitic growth or the aggregation of nanoparticles generated under local high supersaturations in the static conditions. The zigzag appearance is attributed to multiple twinning along the (100) plane.^27,28^ Raman spectroscopy revealed peaks at 41, 71, and 108 cm^–1^ that identified the crystals as β-AG,^29^ and no other guanine polymorphs or guanosine crystals were seen. (Figure 1c) In contrast, only a few larger, plate-like crystals (50–100 μm × 10 μm) formed after 4 days when either the PNP or phosphate buffer were eliminated (Figure S1a). These were identified as reprecipitated guanosine crystals (Figure S1b), demonstrating that both reagents are essential to the production of guanine. In comparison, β-AG crystals precipitated at high pH using published methods^30^ comprised bundles of rods that were over 100 μm in length (Figure S2).

**Figure 1:**
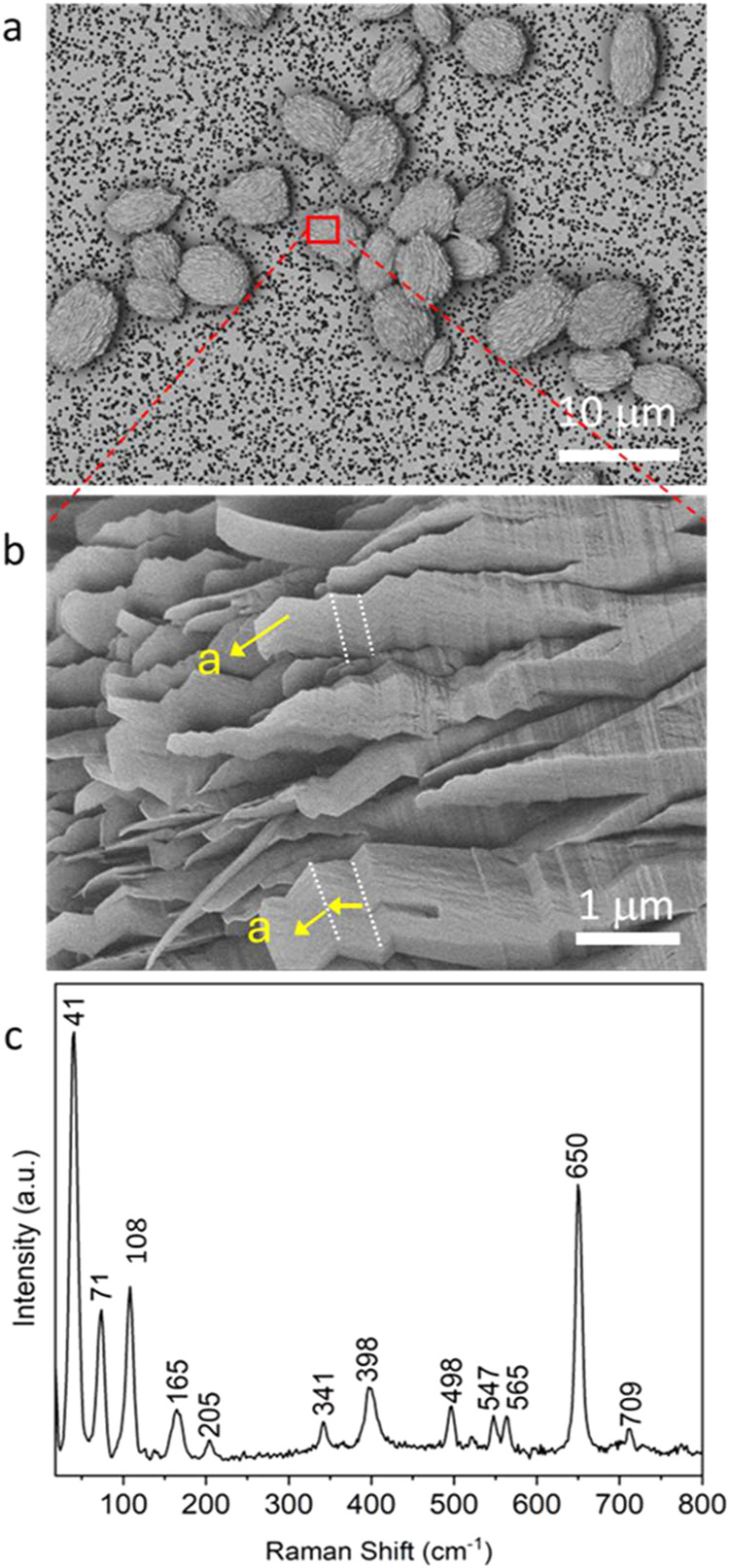
(a) SEM image of spherical β-AG particles and (b) a high-magnification image of the red box in (a) that shows that the particles comprise zigzag-shaped crystals. The white dashed lines indicate twinning planes. (c) A Raman spectrum of the crystals in (a).

### Influence of Substrate Concentrations

The influence of the enzyme kinetics on the formation of guanine crystals was investigated by varying the concentrations of PNP, guanosine and phosphate. Looking first at PNP, β-AG crystals were also produced using 0.5 units of PNP in 50 mM phosphate buffer at pH 7.2, but plate-like α-AG crystals formed when the PNP concentration was further reduced to 0.2 units (Figure S3). α-AG crystals also formed when the initial guanosine concentration—and thus the rate of production of guanine—was reduced to 0.87 mM, while maintaining 1 unit of PNP (Figure S4).

The influence of the phosphate buffer concentration was investigated while keeping all other conditions unchanged (Figure 2). Buffer concentrations of 10 mM were insufficient to maintain a pH of 7.2. Concentrations of 50 mM were sufficient and 5–10 μm spherulitic polycrystalline particles comprising zigzag β-AG crystals formed after 2 days (Figure 1a). Similar crystals also formed at 75 mM (Figure 2a) and 100 mM (Figure 2b), but were accompanied by plate-like crystals of α-AG decorated with smaller zigzag β-AG crystals. The crystal polymorphs were confirmed using Raman spectroscopy (Figure 3b,3c) and powder X-ray diffraction (PXRD). PXRD identified β-AG alone at 50 mM and 75 mM phosphate buffer at the end of the reaction, and a mixture of α-AG and β-AG in 100 mM buffer (Figure 2c). No further changes were observed when concentrations were increased above 100 mM.

**Figure 2:**
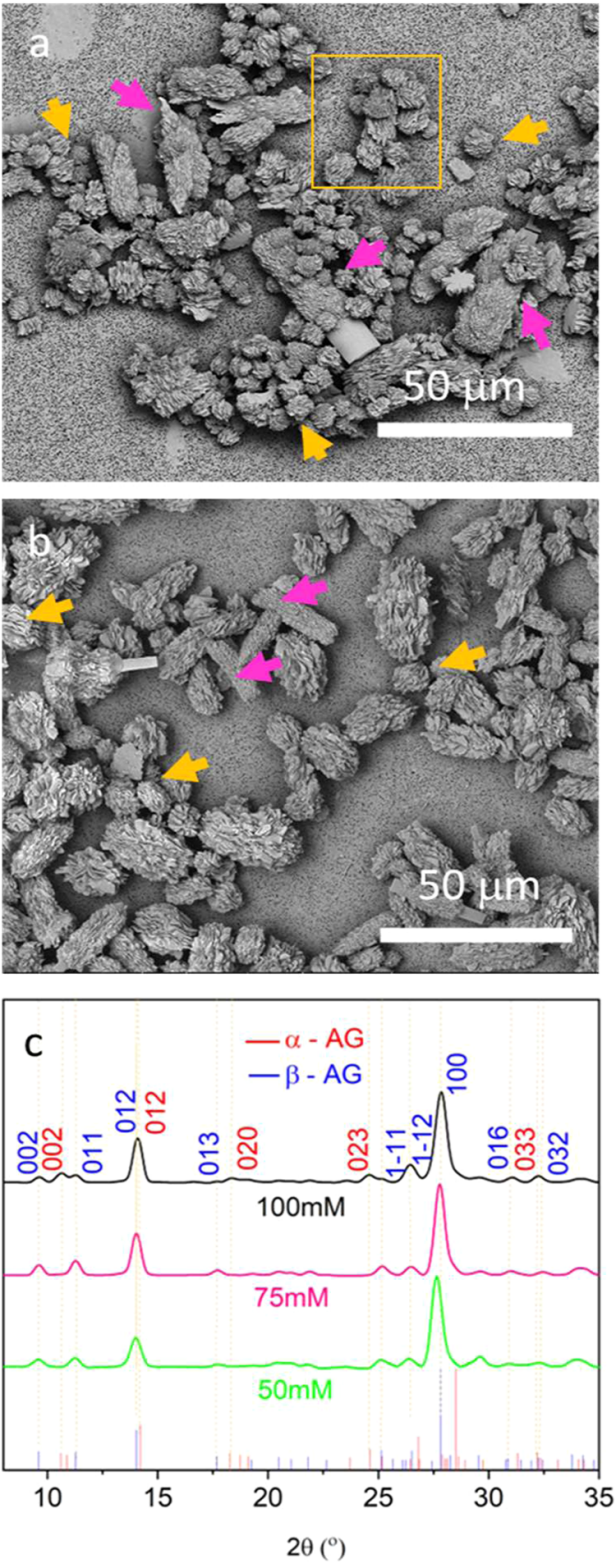
(a, b) SEM images of guanine crystals obtained after 2 days in (a) 75 mM and (b) 100 mM phosphate buffers and standard conditions. The elongated crystals are α-AG plates decorated by zigzag β-AG crystals (pink arrows). Many spherical β-AG particles (yellow arrows) are also seen in (a, b). (c) PXRD patterns of guanine crystals synthesized in the presence of 50, 75, and 100 mM phosphate buffers.

**Figure 3:**
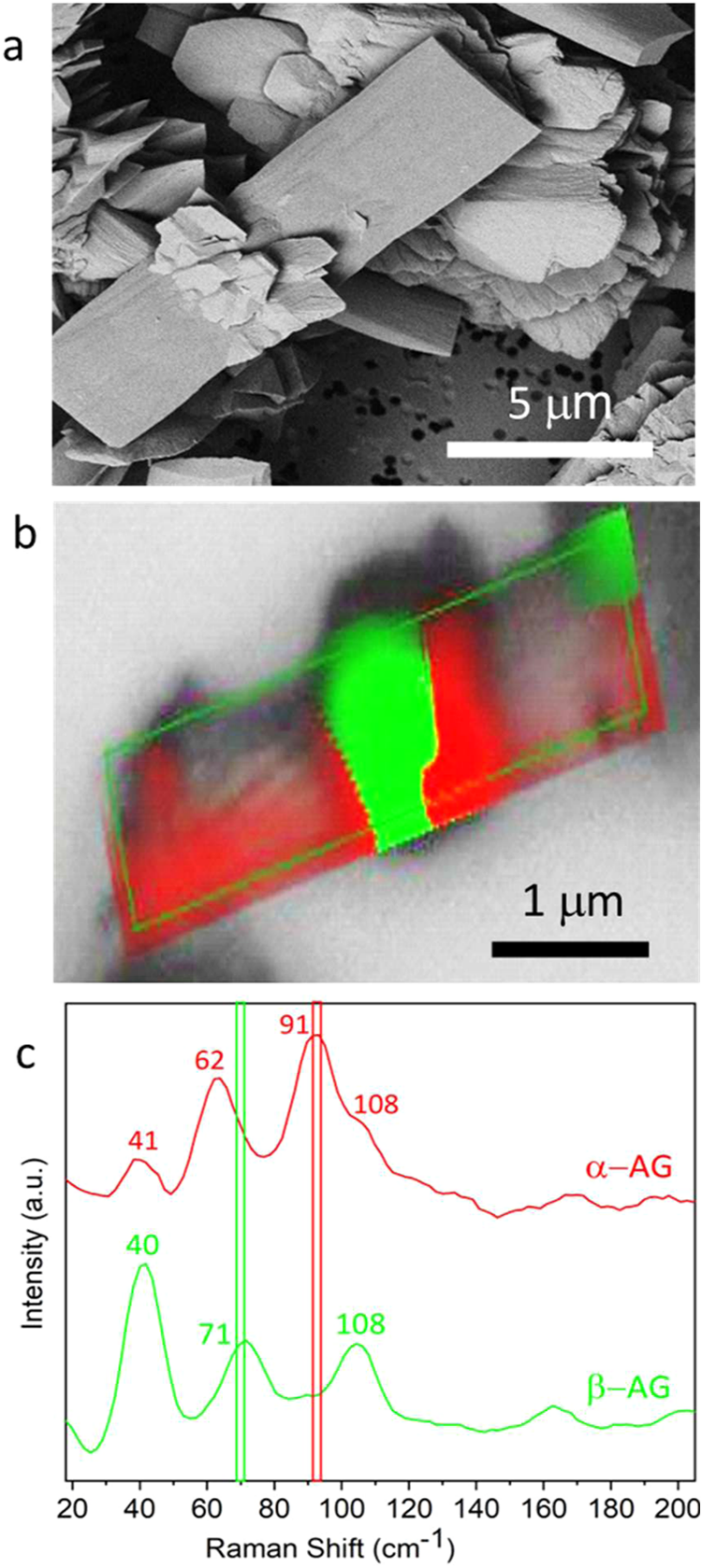
(a) A SEM image, (b) Raman map (green is β-AG and red is α-AG), and (c) Raman spectra of the guanine crystal shown in (b), formed in 100 mM buffer after 12 h.

The development of the crystals at 50, 75, and 100 mM phosphate buffers was followed over time. Small aggregates of zigzag β-AG crystals formed within the first 12 h in the 50 mM buffer and subsequently grew into 10–20 μm spherical particles (Figures 4a and S5). In contrast, large plate-like α-AG crystals appeared within 12 h in the 75 mM and 100 mM buffers but did not grow further with extended reaction times (Figure 4b, 4c). All crystals that formed after 12 h were β-AG and these initially grew around the middle of the α-AG plates, before completely covering their surfaces (Figure 4b, 4d, 4e). Many spherical particles comprising smaller β-AG zigzag crystals were also observed after 30 h (Figure 4b, 4d, 4e). The proportion of plate-like α-AG crystals was greater in the 100 mM as compared with the 75 mM buffer (Figure 2). These results show that the α-AG crystals act as templates for the nucleation of β-AG, which can be attributed to their similar monoclinic structures and closely related lattice parameters.^29,31^ Specifically, α-AG has lattice parameters of *a* = 3.56 Å, *b* = 9.65 Å, *c* = 18.45 Å and β = 118.5° (space group *P*12_1_/*c*1), while β-AG possesses lattice parameters *a* = 3.59 Å, *b* = 9.72 Å, *c* = 18.34 Å and γ = 119.5° (space group P112_1_/b).^27,29^

**Figure 4:**
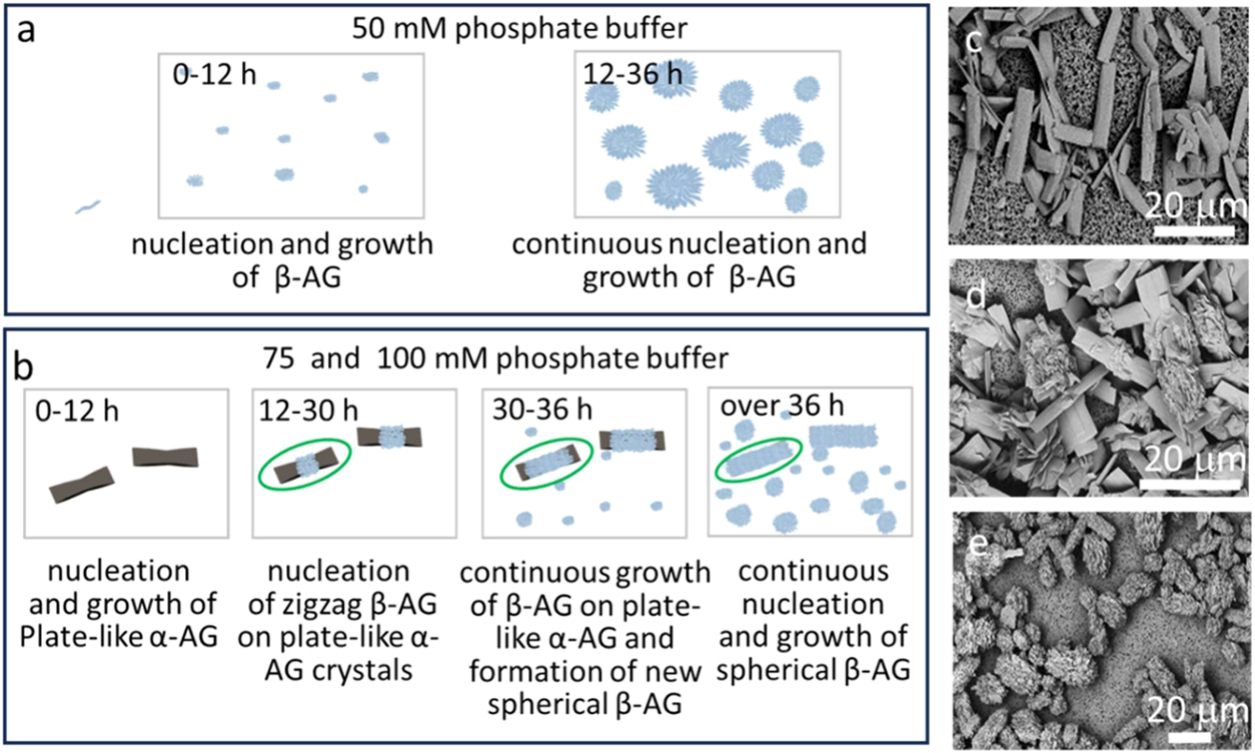
Schematic diagrams illustrating (a) the formation of pure β-AG crystals in 50 mM phosphate buffer over time, and (b) crystallization in 75 and 100 mM phosphate buffer over time. The crystal circled in green is a mixed-phase crystal. (c–e) SEM images of crystals precipitated in 100 mM phosphate buffer after (c) 12 h, (d) 30 h, and (e) and over 36 h of reaction time.

### Polymorph Changes with Reaction Rate

Further insight into the influence of the phosphate buffer concentration on the guanine crystal polymorph was obtained by evaluating the relationship between the rate of the reaction and the polymorph produced. The kinetics of the reaction were characterized using HPLC. In the standard condition with 50 mM buffer, the guanosine concentration fell sharply over the first 30 h, reducing from 1.6 mM to 0.07 mM at an average rate of 53 μM h^–1^ (Figures 5a and S6). The rate then reduced to 3.9 μM h^–1^ after 30 h until all the guanosine had been consumed. The initial conversion rate (over 0–6 h) was dependent on the buffer concentration, being 48 μM h^–1^ at 50 mM, 26 μM h^–1^ at 75 mM and 21 μM h^–1^ at 100 mM (Figure 5b). Little dependence was seen over longer periods, where the conversion rate was 10–16 μM h^–1^ for all conditions. 70, 53 and 39% conversion of guanosine to guanine was recorded after 24 h in 50, 75 and 100 mM phosphate buffers respectively.

**Figure 5:**
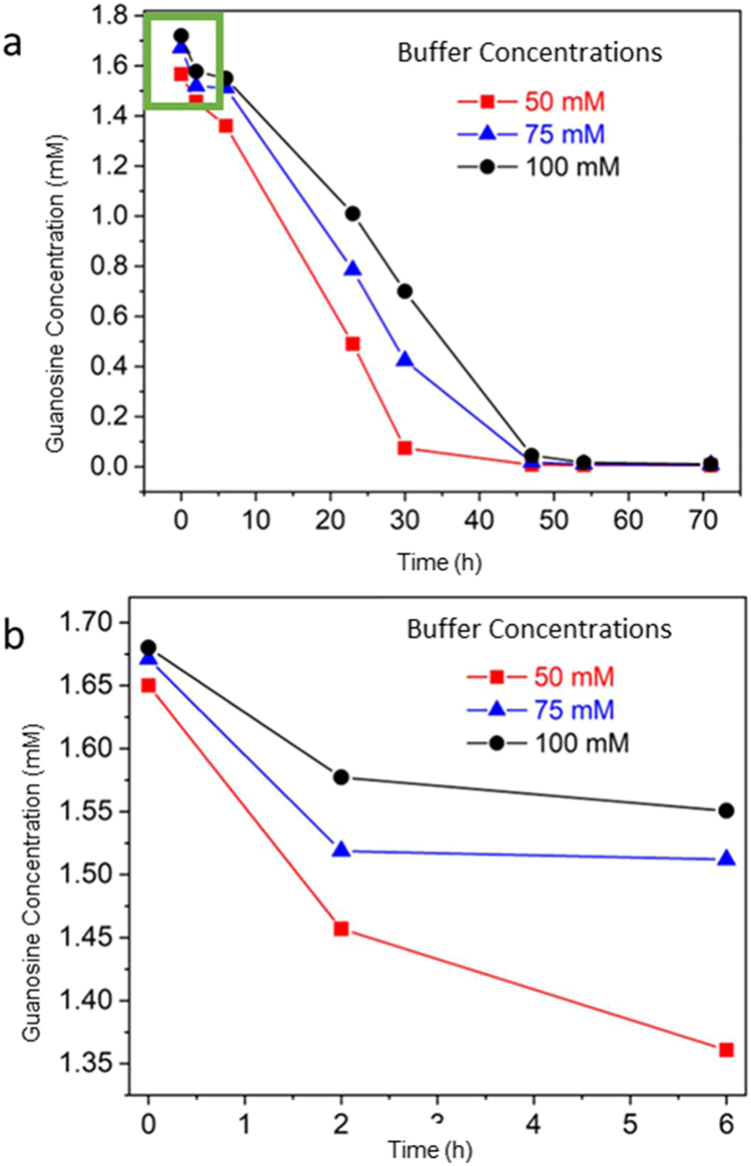
(a, b) Graphs showing the change of guanosine concentration over time at different phosphate buffer concentrations (50, 75, and 100 mM) as measured using HPLC. (b) The change in concentration of guanosine over 0–6 h, delineated by a green box in (a).

These results suggest that stable α-AG crystals preferentially form when there is a slower increase in supersaturation. This trend was also confirmed using ^31^P nuclear magnetic resonance (NMR) to follow the changes in concentrations of phosphate and the ribose-1-phosphate byproduct of the conversion (Figure S7). These data also show that α-AG is produced under conditions in which guanosine is slowly converted to guanine.

The influence of the rate of the reaction on the crystal polymorph was further explored by stirring solutions to enhance the reaction rate. HPLC measurements carried out when 20 mL of the standard reaction solution (50 mM phosphate concentration with 1 unit PNP) was magnetically stirred showed that the average conversion rate of guanosine to guanine within the first 5 h was 2.6 times greater than in static solutions (Figure 6a) and the reaction terminated within 1 day, as compared with 2 days when the reaction was not stirred. Raman spectroscopy and PXRD confirmed that the crystals produced in stirred solutions were β-AG, as was observed in unstirred solutions (Figure 6b, 6c). The same results were obtained from solutions containing 75 and 100 mM phosphate. This contrasts with unstirred conditions when α-AG crystals form within the first 12 h and a mixture of α-AG and β-AG crystals are present after 2 days.

**Figure 6:**
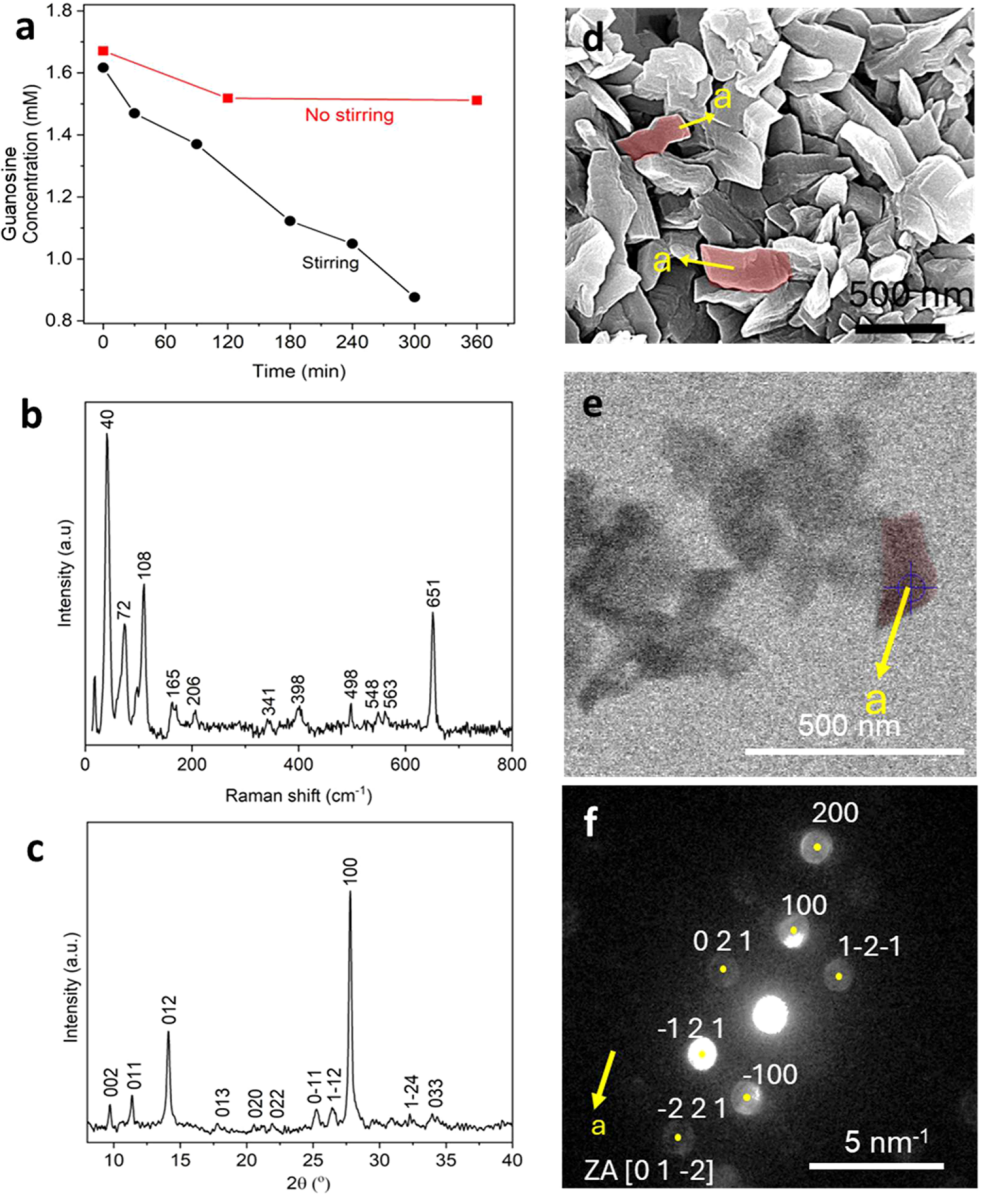
(a) Graph showing the change of guanosine concentration over time with and without stirring under standard solution conditions (50 mM phosphate buffer, 1 U PNP) as measured using HPLC – UV. (b) Raman spectrum and (c) PXRD pattern of the β-AG crystals obtained with stirring under standard conditions. (d) SEM image, (e) STEM image, and (f) the corresponding STEM diffraction pattern of β-AG crystals precipitated under standard conditions with stirring.

Notably, the β-AG crystals formed were individual zigzag-shaped particles that were visibly distinct from the larger spherical aggregates observed after 1 day under unstirred conditions (Figures 6d and S8). This confirmed that the crystals were β-AG and elongated along the *a*-axis (Figure 6e, 6f).

### Effects of Additives on Morphology

Having developed a method for forming guanine crystals under physiological conditions, the effect of organic additives on crystal morphologies was explored. While synthetic β-AG crystals are prismatic and elongated along the *a*-axis (π-stacking direction), (Figure 6d–f), their biogenic counterparts often form as thin plates elongated along the *c*-axis (hydrogen-bonding direction) (Figures 7 and S9). Biogenic guanine crystals typically contain about 20% hypoxanthine,^32^ and it has been hypothesized that purine additives may inhibit growth along the π-stacking direction (the *a*-axis), leading to the formation of plate-like crystals. Poly­(vinylpyrrolidone) (PVP) has also been used to generate plate-like β-AG crystals in DMSO and formamide.^33^ To explore this possibility in our system, we tested three different types of additives, amino acids (tryptophan and histidine), dyes (rhodamine 6G and orange G), purines (hypoxanthine, uric acid) and polymers. These share common features of planar structure with aromatic conjugation which potentially inhibit the growth along the stacking directions (*a*-axis), therefore promoting growth along the hydrogen bonding direction. Polymeric structures are chosen to increase inhibiting efficacy due to multiple binding sites. The chemical structures of these additives are shown in Figure S10.

**Figure 7:**
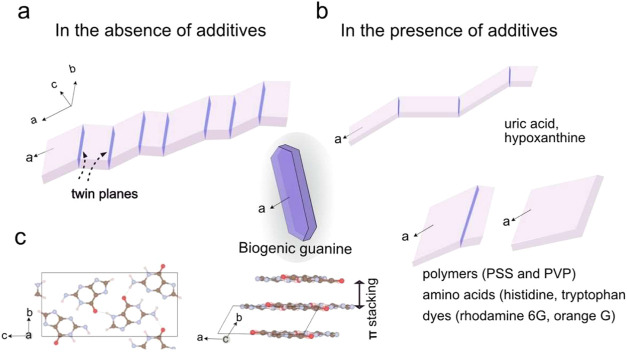
Summary of the morphological variation of β-AG crystals obtained in solution: (a) In the absence of additives, the crystals exhibit multiple twinning on the (100) plane. (b) In the presence of additives, the degree of twinning is reduced. Purine additives promote elongation along the *a*-axis (π-stacking direction), while dye, amino acids, and polymer additives result in shorter crystals, as discussed in detail in the main text. (c) β-AG crystal structures showing the hydrogen-bonding plane viewed along the *a*-axis and the π-stacking direction viewed along the *c*-axis. The shaded inset illustrates a representative morphology of biogenic β-AG crystals, noting that their orientation is perpendicular to that of the synthetic crystals.

The effects of these additives on the morphologies of the guanine crystals were investigated with stirring and additive concentrations of 0.5–1 mg mL^–1^. β-AG crystals precipitated in the presence of hypoxanthine (Figure 8a,8d) and uric acid (Figures S11b and S12a) were slightly elongated along the *a*-axis, and occasionally twinned along (100) planes. This suggests that these molecules weaken hydrogen bonding interactions, causing an elongation of the guanine crystals along the π-stacking direction.^34^ β-AG crystals exhibited a reduced aspect ratio in the presence of PSS (Figure 8b,8e), while PVP, in contrast, yielded thick, plate-like α-AG crystals that were elongated along the *a*-axis (Figures S11c and S12a). Rhodamine 6G altered both the crystal morphology and polymorph, generating α-AG crystals that were shorter along the *a*-axis and slightly elongated along the *b*-axis, giving square or rectangular shapes (Figure 8c,8f), while orange G had no significant effects (Figures S11d and S12b). The amino acids tryptophan and histidine also had little effect on the morphology or polymorph of the crystals (Figures S11e,f and S12a). The activity of these additives was also explored in aqueous solution at high pH, where the β-AG polymorph was produced, and no change in morphology was observed (Figures S13 and S14).

**Figure 8:**
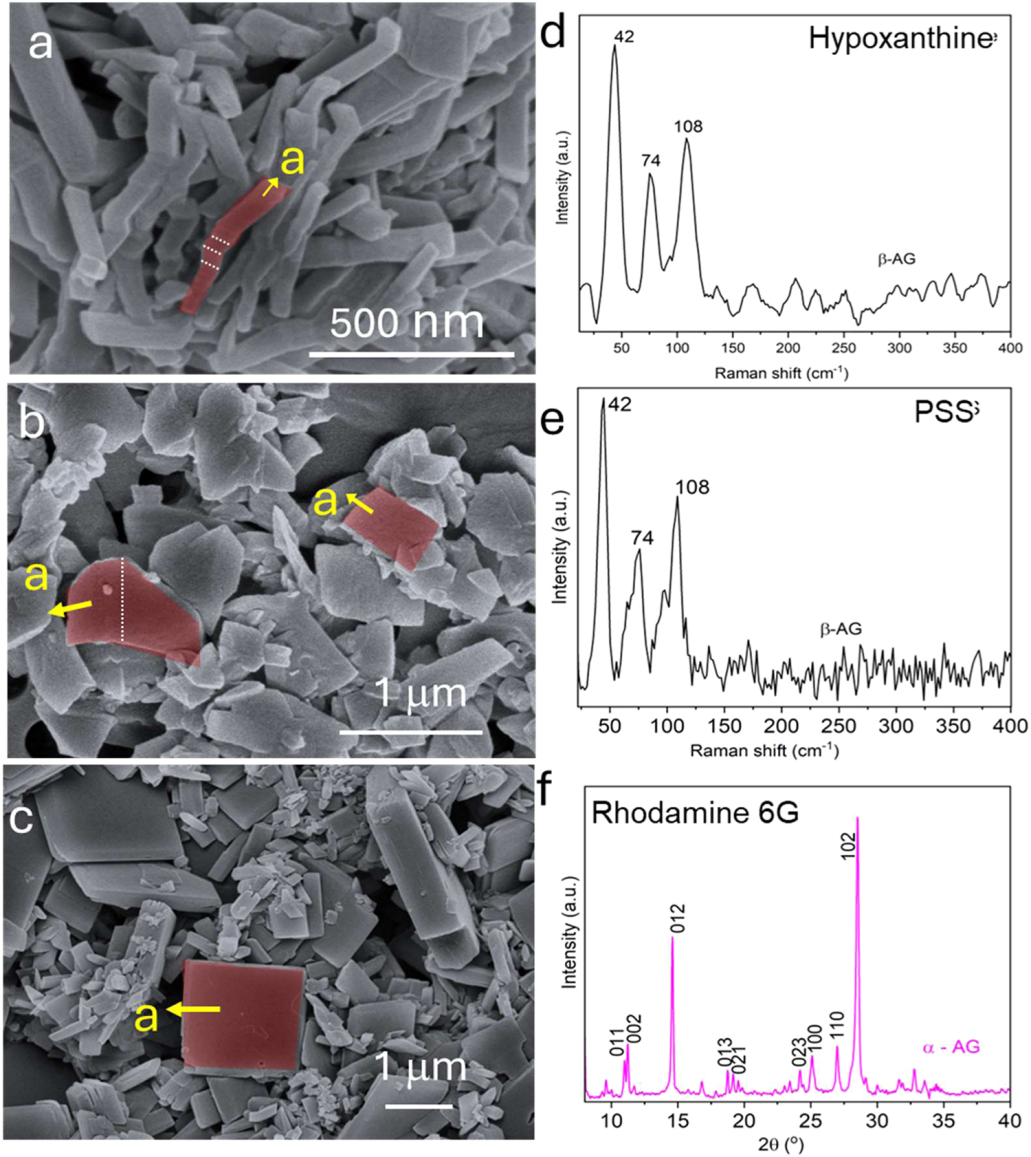
SEM images of guanine crystals obtained after 1 day with 1 unit of PNP and 50 mM phosphate buffer under stirring, with 0.5 mg/mL addition of (a) hypoxanthine, (b) PSS, and (c) rhodamine 6G. (d, e) Raman spectra of guanine crystals synthesized in the presence of hypoxanthine and PSS, respectively. (f) XRD pattern of guanine crystals synthesized in the presence of rhodamine 6G. The white dashed lines indicate twinning planes.

## Discussion

This body of work demonstrates that the production of β-AG at physiological pH is governed by the rate of guanosine conversion to guanine, which in turn dictates the rate of change of the solution supersaturation. This is evidenced by (i) the formation of pure β-AG at high initial concentrations of guanosine and enzyme, as compared with pure α-AG at low initial concentrations; (ii) an acceleration in the formation of β-AG crystals when the solution is stirred; and (iii) the initial formation of α-AG crystals followed by β-AG crystals at higher phosphate concentrations (75 and 100 mM) when the rate of guanosine conversion is lower.

These findings suggest a possible relevance to guanine crystallization in biology, indicating that organisms may generate β-AG crystals by controlling the supply of guanine molecules to the localized environment in which the crystals form. High supersaturations can be created in these small, ultraclean environments, and crystallization could be triggered as required, possibly by a change of pH.^13^ Notably, the limited solubility of guanine in water at neutral pH^26^ has made it challenging to study the crystallization of guanine under biomimetic conditions. β-AG crystals have been previously formed by dissolving guanine in high or low pH solutions and then rapidly changing the pH to create a supersaturated state.^13,14^ They have also been precipitated at the air/water interface by exposing an acidic solution of guanine to ammonia gas and causing a local increase in pH,^15^ and in a mixed solution of alkaline water and formamide (at 40 °C) in which the guanine has a much higher solubility, and initiating crystallization by a pH change. Hydrated amorphous guanine also converted to β-AG crystals in organic solvents including DMSO, formamide, and DMF, but to α-AG in water.^33^ All of these approaches induce a rapid change in supersaturation, causing crystallization to occur at the higher supersaturations that favor metastable β-AG.

Recent cryo-electron microscopy studies of zebrafish larvae^11^ and juvenile scallop eyes^10^ have provided some insight into the mechanisms by which organisms create intracellular guanine crystals and have suggested that the β-AG nucleates within preassembled fibrillar scaffolds in chromatophores, and that multiple nucleation events occur within the scaffolds. Interactions between specific amino acids and guanine molecules, or between hydrophobic domains in the fiber and the hydrogen-bonded guanine layers may direct the crystal orientation and morphogenesis. Such molecular recognition is unlikely to select the crystal polymorph, however, as both α-AG and β-AG have similar structural units comprising extended hydrogen-bonded layers.

Our system also gave an opportunity to explore the effect of organic additives on the polymorph and morphology of guanine crystals at physiological pH. β-AG crystals (i.e., no change in polymorph) were produced with most of the additives, but PVP and rhodamine 6G induced a change from β-AG to α-AG. Notably, changes in crystal morphologies were generally minor in both acidic and basic pH regimes. These results are interesting when considered in light of a recent study of the formation of guanine crystals in zebrafish, where the crystals are actually cocrystals of guanine and hypoxanthine. Notably, a close correlation between the chemical composition and morphologies of the crystals,^25,28^ leading to suggestions that interactions between the growing crystals and hypoxanthine and uric acid molecules leads to a morphological change. Our observation that these molecules consistently induce elongation along the *a*-axis reinforces the idea that hypoxanthine effectively modulates hydrogen bonding and crystal growth dynamics in this direction.

Our work also offers insight into the stability of metastable β-AG crystals, where there is a lack of consensus in the literature regarding the stability and mechanism of transformation of β-AG to α-AG crystals.^29,32,33,35^ The stability of the synthesized β-AG crystals was investigated in air, DI water, 50 mM HEPES buffer (pH 7.2), and in the mother solution where the enzymatic reaction occurred. The β-AG crystals remained unchanged in the mother solution and in air for at least 20 days, but partially transformed to α-AG in DI water and HEPES buffer after 18 h (Figure S15). β-AG crystals synthesized at high pH also remained stable in air for at least 20 days.

In contrast, β-AG crystals extracted from dinoflagellate were reported to rapidly convert to α-AG^29^ and β-AG crystals present in spider integument partially converted to α-AG,^32^ where the latter was proposed to occur via a solid-state transformation. β-AG crystals from fish scales showed greater stability, where the observed partial transformation to α-AG was attributed to conversion of an amorphous phase.^35^ However, with the information available in these articles, it is often not clear how the crystals were extracted, and thus whether conversion could have occurred by a dissolution/reprecipitation mechanism during extraction. Notably, most β-AG crystals synthesized in organic solvents have been reported to be stable in air and in their mother solutions,^33^ which is in keeping with our observations. This is also indicative of the transformation occurring by a dissolution/recrystallization mechanism.

## Conclusions

This work introduces an effective enzyme-mediated synthesis of guanine crystals that operates at neutral pH and which delivers control over the guanine polymorph by simply tuning the solution concentrations (phosphate, guanosine, and enzyme) or by stirring the reaction solution. Pure β-AG or α-AG crystals were reproducibly synthesized using this approach, despite a difference in solubility of these polymorphs of only 1.2 μM^12^ and a calculated lattice energy difference of 0.39 kcal mol^–1^ (1.63 kJ mol^–1^).^35^ This suggests that organisms could potentially influence the polymorph of guanine crystals by modulating the rate of crystallization. Our new strategy also enables the influence of biologically relevant organic molecules on guanine crystallization to be investigated at physiological pH. That only minor effects were observed in our experiments suggests that the environment in which the crystals form may play the salient role in generating the plate-like morphologies often observed in organisms. This work lays the groundwork for further exploration of biogenic crystallization mechanisms, focusing on the effects of biologically relevant soluble additives and confinement strategies to enhance control over crystal morphology, polymorph, and orientation.

## Supplementary Material



## Data Availability

The data that support the findings of this study are openly available in the Research Data Leeds Repository at https://doi.org/10.5518/1374.^1^

## Notes

Additional experimental details, materials, synthesis, characterization, SEM and TEM analysis, RAMAN spectroscopic analysis, ^31^P NMR spectra of the reaction solution, chemical structures of additives used (Figures S1–S15) (PDF)
