# Supplementary material for: Guanine Crystal Formation at Physiological pH
Source: Cryst Growth Des. 2025 May 30;25(12):4316–24. doi: 10.1021/acs.cgd.5c00205 (PMC12186259; doi:10.1021/acs.cgd.5c00205)
Supplement: Supplementary file 1 [file cg5c00205_si_001.pdf]

Guanine Crystal Formation at Physiological pH

Bidisha Tah Roy, Lukas Jorin Hasselt, Ross Young, Zijiang Yang, Jeanine Williams, Johanna M. Galloway, Alex Heyam, Yi-Yeoun Kim and Fiona C. Meldrum**

School of Chemistry, Woodhouse Lane, University of Leeds, Leeds, LS2 9JT, United Kingdom

1. Materials

Guanosine ($C_{10}H_{13}N_5O_5$), potassium phosphate dibasic (K_2HPO_4), potassium phosphate monobasic (KH_2PO_4), enzyme purine nucleoside phosphorylase (PNP) bacterial, expressed in *E. coli*, sodium azide (NaN_3), 2,2-dimethyl-2-silapentane-5-sulfonate sodium salt (DSS), ammonium bicarbonate (NH_4HCO_3), 2-[4 -(2 -hydroxyethyl) piperazin-1-yl] ethanesulfonic acid (HEPES), trifluoroacetic acid (TFA), uric acid, hypoxanthine, polyvinylpyrrolidone (PVP, Mw 1,300k), polystyrene sulfonate (PSS, Mw 70k), rhodamine 6G, orange G, L-tryptophan, were purchased from Sigma Aldrich. L-histidine monohydrochloride monohydrate was purchased from Fluka. All chemicals employed were analytical reagents with a purity of 99% and were used without additional purification. Double distilled water was prepared using Millipore Milli-Q EQ-7000 system (< 5 p.p.m. total organic content (TOC) and 18.2 MΩ.cm))

Purine nucleoside phosphorylase (PNP) enzymes can be obtained from many different sources, human, calf spleen, human erythrocytes and E. coli, and their structures and properties vary depending on the source. In this work the PNP used was DeoD-type, homohexamer enzyme 2.4.2.1 subunit Mr = 26 KDa (239 sequence), expressed in *E. coli*, which is less specific to substrates and more robust under a wide range of conditions. It shows a broad pH activity ranging from 6 – 8.6, with optimal pH 7-8. The structure and sequences of PNP were reported[1]

2. Synthesis

2.1 Enzyme preparation

250 mg of PNP was dissolved in a 10 mL of 50 mM ammonium bicarbonate solution. The concentration of PNP was determined using UV-Vis spectroscopy (absorbance 280 nm) with a NanoDrop spectrophotometer (Thermo Scientific). Subsequently, the solution was divided into several 2 mL

Eppendorf tubes, each containing 4 units of PNP. These solutions were frozen overnight and then freeze-dried overnight using a LABCONCO FreeZone 1 litre Benchtop Freeze Drier Systems. The The resulting solid was stored in a freezer at -18°C until use. The freeze-dried PNP was dissolved in a 50 mM phosphate buffer solution (pH 7.2), and the concentration of PNP was measured again using the NanoDrop Lite spectrophotometer. Finally, 1 unit of PNP (unless specified) was used in each experiment within a 22 mL glass vial containing 20 mL of reaction solution (i.e., PNP concentration = 5 mg mL^{-1}). 1 unit of PNP is the amount of enzyme that converts 1 mM of guanosine per min in the presence of phosphates.

2.2 Enzymatic guanine crystallization

Phosphate buffer solutions were prepared using different ratios of K_2HPO_4 and KH_2PO_4 to give concentrations of 50 mM, 75 mM or 100 mM while maintaining the pH at 7.2. The solutions were kept in a fridge at 4°C. 100 mL of 1.75 mM guanosine solution was prepared by dissolving it in 500 mg L^{-1} (1.75 mM) in the phosphate buffer solutions pH 7.2. The guanosine solution was further sonicated in an ultrasonic bath (Clifton SW6H 6-liter) for 20 mins to ensure full dissolution. The desired amounts of PNP enzyme (0.2, 0.5, and 1 unit) were subsequently added to 20 mL of the guanosine solution in a glass vial. To inhibit bacterial growth, sodium azide (1.5 mM) was added to each solution, and the vials were sealed with lid until used. Purine additives (hypoxanthine and uric acid), polymer additives (PSS and PVP), amino acids (L-histidine monohydrochloride monohydrate and L-tryptophan), and dyes (rhodamine 6G and orange G) were added to the guanosine solution at concentrations of 0.5-1.0 mg mL^{-1}. The reactions were conducted at room temperature for maximum 15 days. The same procedure was used for the synthesis with stirring, but the reaction took place on multi-point magnetic stirrer at 120 rpm. The products were filtered using a hand pressured filtration unit (Millipore isopore polycarbonate membrane, 0.22 μm pore size and 13 mm in diameter) and rinsed with water and dried under air.

2.3 Synthesis of guanine crystals at high pH

A solution containing 3.3 mM guanine was prepared in 1 M NaOH. Subsequently, 1 M HCl was added dropwise until the pH reached 11. Upon reaching this pH level, the solution became turbid, and the suspension was promptly collected via filtration, and rinsed with water and dried under air.

2.4 Transformation of guanine in various solutions and air

The synthesized guanine crystals were incubated in 200 µL of DI water, mother solution and 50 mM HEPES buffer solution at pH 7.2 for approximately 18 h. They were also exposed to air for at least 3 weeks. The crystals were then collected by filtration, rinsed with water, and air-dried as described previously.

3. Characterization

3.1 Scanning electron microscopy (SEM)

Samples were placed onto track etched membranes or silicon wafers, and were then mounted on SEM stubs using carbon adhesive discs. The samples were coated with a 4 nm iridium layer prior to imaging. Images were obtained using an FEI Nova 450 NanoSEM using a circular backscatter detector (CBD) and through-the-lens detector (TLD) at 3 or 5 keV.

3.2 Low wavenumber RAMAN microscopy

Samples were dried onto a track-etched membrane or a clean silicon wafer, and were then imaged using an Olympus optical microscope. Raman spectra were collected using a Horiba Labram HR Evolution Raman microscope equipped with a solid-state laser with wavelength 532 nm with respective ultra-low frequency (ULF) modules allowing measurements in the sub 100 cm^{-1} region, operating at a laser power of 1% - 3% of 50 W. Each accumulation of data was performed for 20 seconds with three times of accumulation to enhance the signal and reduce noise. The acquired data were subsequently analyzed using LabSpec6 software.

3.3 Powder X-ray diffraction (PXRD)

Crystals formed in unstirred reaction solutions were collected by filtration and were then placed in 1 mm diameter borosilicate glass capillary tubes with wall thicknesses of 10 µm. Diffraction data were collected using hybrid photon counting 2-D detector using a Rigaku XtalLAB Synergy Custom instrument with a MM007-HF (Cu) microfocus rotating anode and VariMAX high X-ray optics flux source. The detector was set at 80 mm, with an operating voltage of 40 kV and a current of 30 mA. The exposure time was 15 minutes, covering a 2θ range from 0 to 38 degrees. The XRD data were acquired and processed using Origin software.

Crystals formed in stirred reactions were dried onto clean silicon wafers and diffraction data were recorded using a Brucker Phaser D2 diffractometer (Cu Kα source, 1.5406 Å), equipped with a LynxEye detector configured in the Bragg-Brentano geometry with scanning range 5 - 50° 2θ. The data was processed using DiffractEVA software.

3.4 Ultraviolet-visible (UV-Vis) spectroscopy

Data were recorded using a NanoDrop Lite UV-Vis spectrophotometer using 2-3 μL of enzyme solution. Each measurement was repeated three times.

3.5 High performance liquid chromatography (HPLC)

HPLC analysis was used to follow the conversion of guanosine to guanine in solution over time (0 – 72 hours). 200 μL volumes of the reaction solutions were extracted at different time points and 1 μL aliquots were directly injected into the column. The analysis was performed using an Agilent 1290 Infinity II HPLC system (Agilent, Santa Clara, USA), with a diode array detector. Chromatographic separations were performed using an Agilent Eclipse XDB-C8 (4.6 x 150 mm, 5 mm) with ambient column temperature. The mobile phase was prepared by mixing equal volumes of 0.1% TFA in water and 0.1% TFA in acetonitrile and used at a flow rate of 0.8 mL/min. The Diode Array Detector recorded the chromatogram at a wavelength of 280 nm. Guanosine has a retention time of 2.0 min under these conditions. For calibration, a standard stock solution was prepared by dissolving 2 mM guanosine in DI water. This was diluted to create a range of standards between 0.25 and 2 mM of guanosine. A linear response correlation coefficient (R) of 0.9998 with variation of 3.5% at 0.16 mM concentration was obtained.

3.6 Nuclear magnetic resonance (NMR) spectroscopy

1.75 mM guanosine solutions prepared with 50 mM, 75 mM and 100 mM phosphate buffers (pH 7.2) were diluted with D_2O to 90% of their initial concentrations. The NMR data were acquired using a 500 MHz Bruker Avance Neo spectrometer equipped with a room temperature probe. The sample temperature was kept at 298 K. ^{31}P spectra were recorded with a 30 degree excitation pulse and inverse gated proton decoupling, with an acquisition time of 2 sec and relaxation delay of 38 sec. ^{31}P spectra were referenced relative to the solvent. The data was processed using Bruker

Topspin by integrating each spectrum within the same ranges. The "time zero" data was obtained before adding the enzyme into the guanosine solution with 50 mM phosphate buffer.

3.7 Scanning transmission electron microscopy (STEM)

The dried guanine crystals were suspended in ethanol. 20 µl drops were transferred onto a TEM grid (formvar-carbon coated copper 300 mesh, EMS, USA) and dried in air. Imaging and recording of diffraction data of guanine crystals was carried out using a Tescan Tensor fitted with the Dectris Direct Electron Detector. The instrument was operated at 100 kV, with a STEM probe beam current of 50 pA and detector angle 2 mrad. The data were collected using Expert PI software.

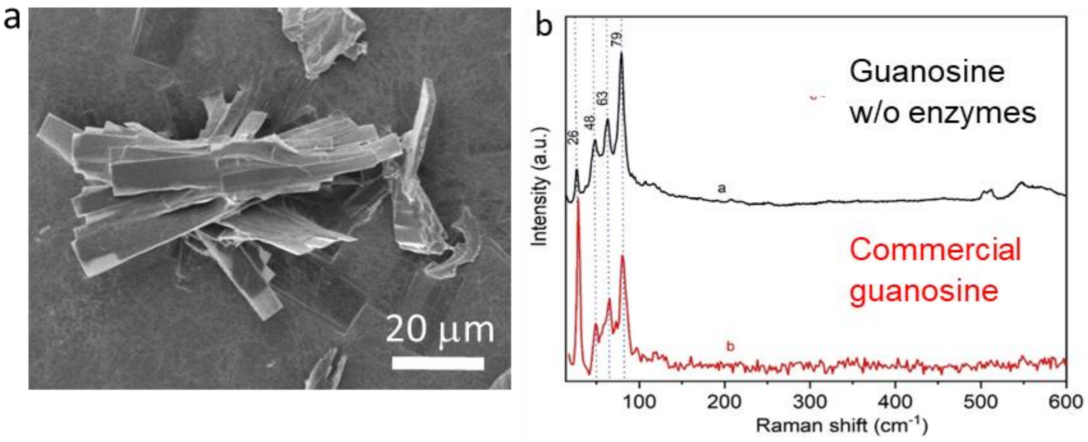

Figure S1. (a) SEM image of plate-like guanosine crystals precipitated in a supersaturated solution of guanosine in the absence of enzyme PNP after 4 days. (b) Raman spectra of the reprecipitated guanosine crystal and commercial guanosine, both showed characteristic peaks at 26, 48, 63 and 79 cm^{-1}.

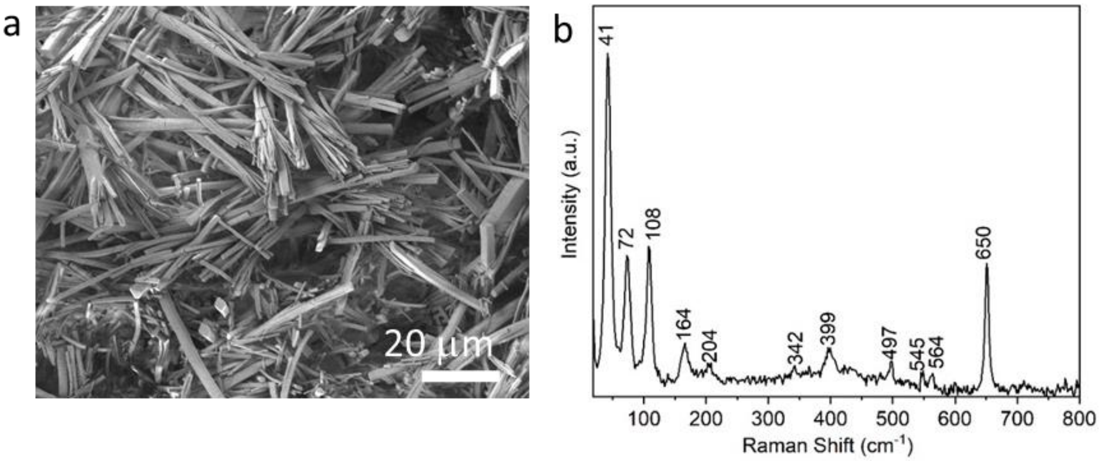

Figure S2. (a) a SEM image and (b) a Raman spectrum of β-AG crystals synthesized at pH 11.

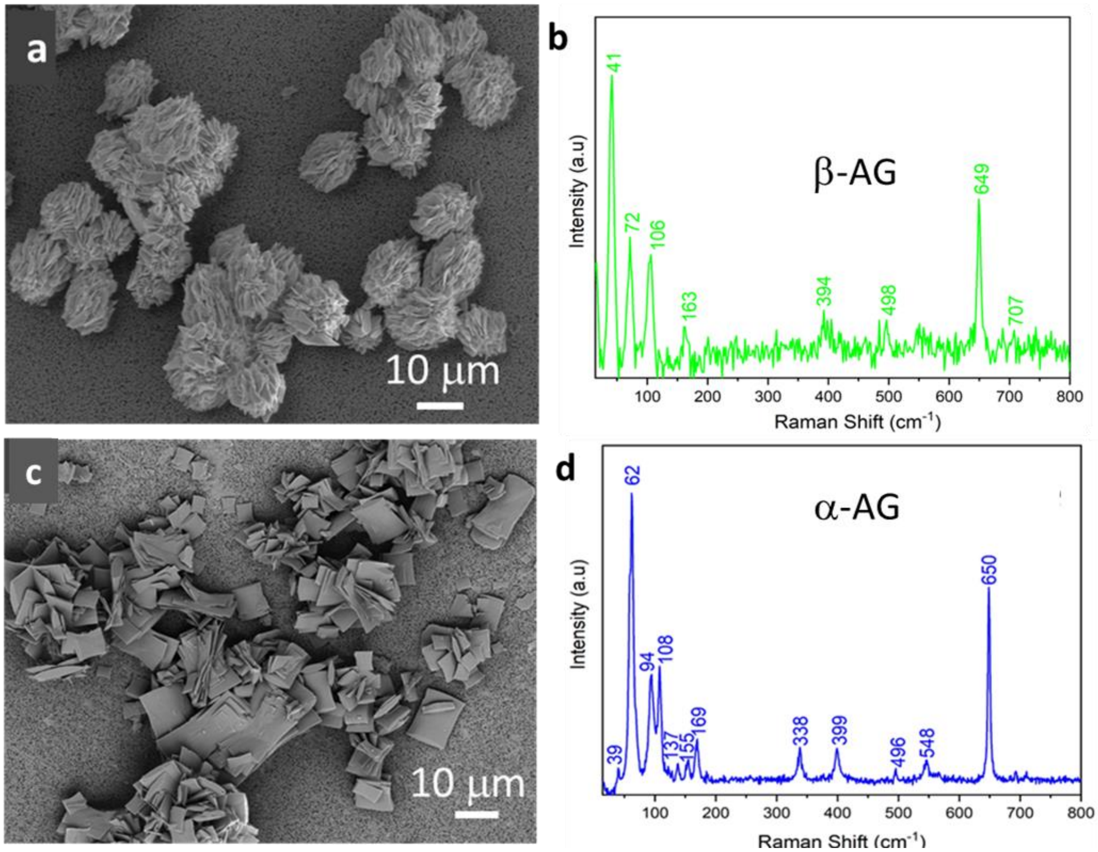

Figure S3: The effects of reduced enzyme concentrations on guanine crystallization. (a and c) SEM images and (b and d) Raman spectra of crystals obtained after 2 days in 50 mM phosphate buffer in the presence of (a and b) 0.5 units of PNP and (c and d) 0.2 units of PNP.

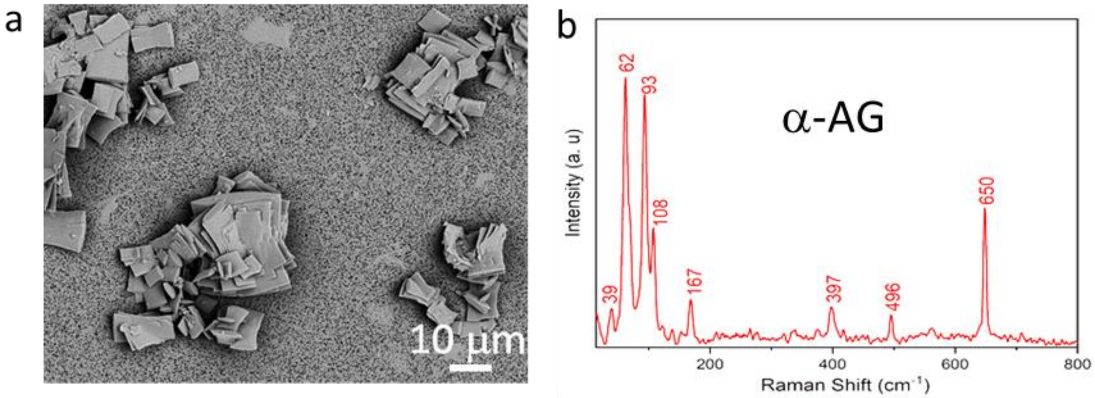

Figure S4. The influence of reduced initial guanosine concentrations.(a) SEM image and (b) Raman spectrum of α-AG crystals obtained after 2 days in the presence of 0.87 mM guanosine in 50 mM phosphate buffer with 1 unit PNP.

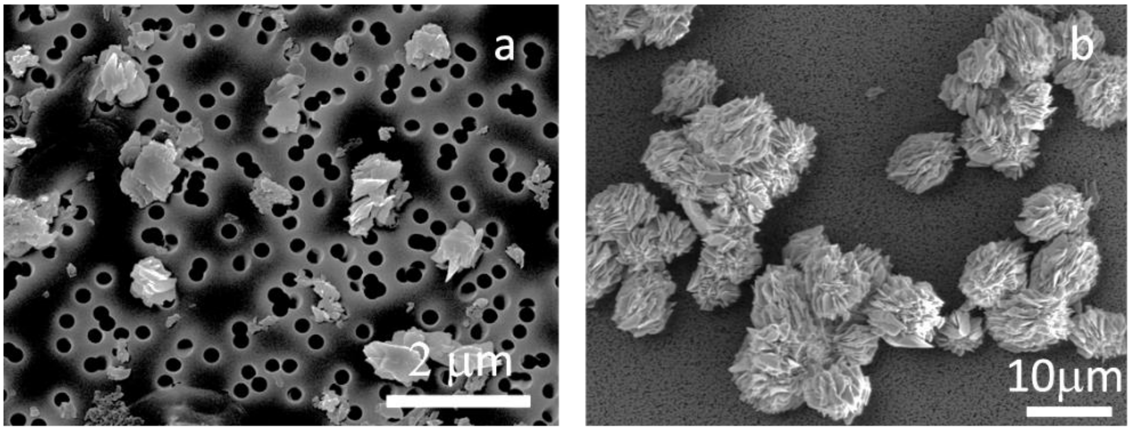

Figure S5: SEM images of β-AG particles consisting of zigzag shaped crystals. (a) 12 h and (b) 36 h of reaction in 50 mM phosphate buffer condition. (note: track-etched membranes were used to collect the guanine crystals)

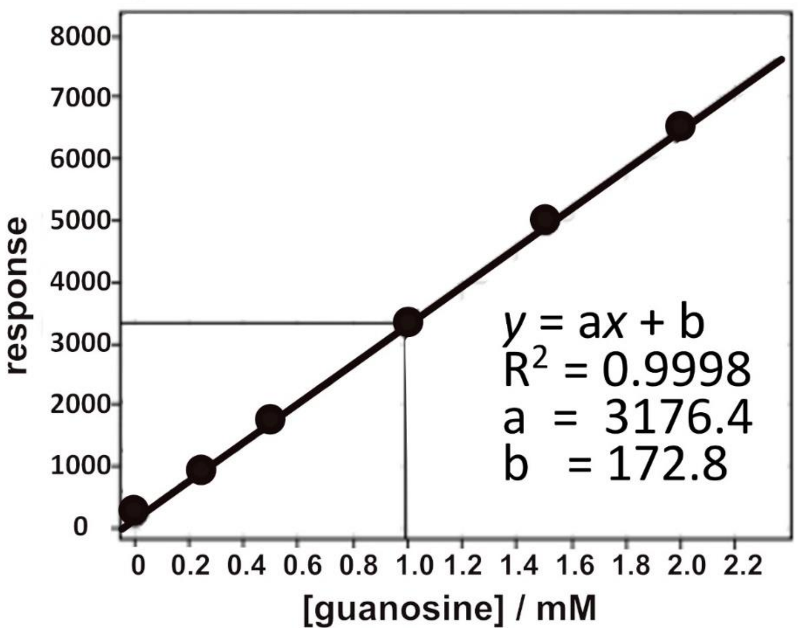

Figure S6. Calibration curve used to ascertain the guanosine concentration based on the HPLC chromatography peak. This established the relationship between the guanosine concentration and the corresponding area response.

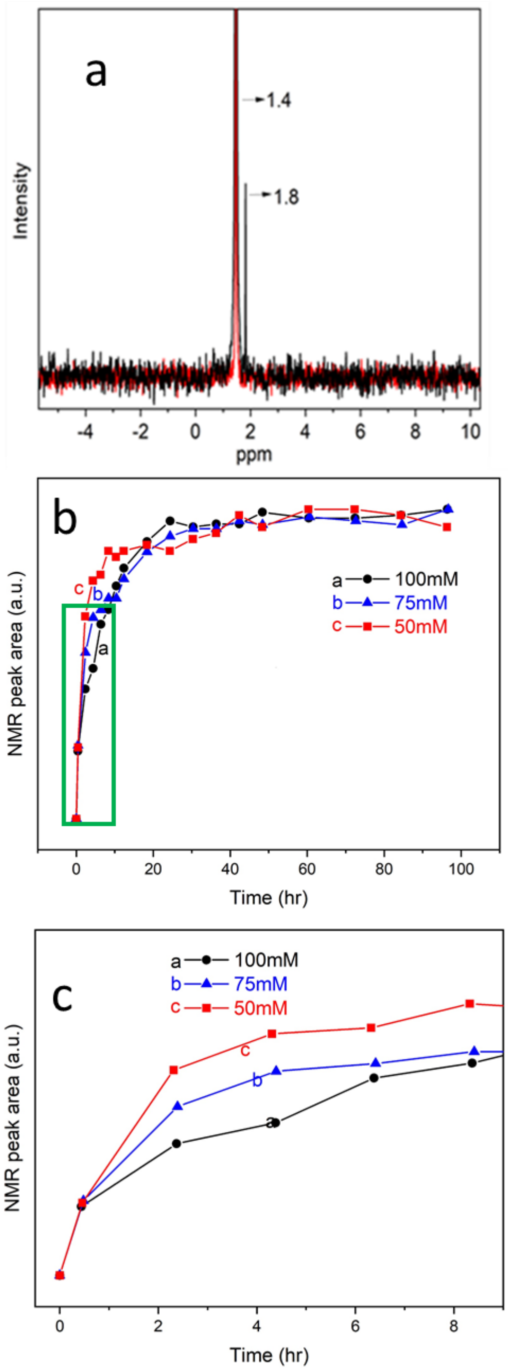

Figure S7. (a) ^{31}P NMR spectra of the reaction solution before adding PNP (red) and after 4 days of reaction in the presence of PNP (black). The 1.4 ppm and 1.8 ppm peaks correspond to phosphorous from phosphate and ribo-1-phosphate respectively. (b) Increase in NMR peak area for ribo-1-phosphate between 0 and 100 h. (c) The peak area of ribose-1-phosphate changes in the early stages of the reaction (0 – 8 h), as indicated by the green box in panel (b).

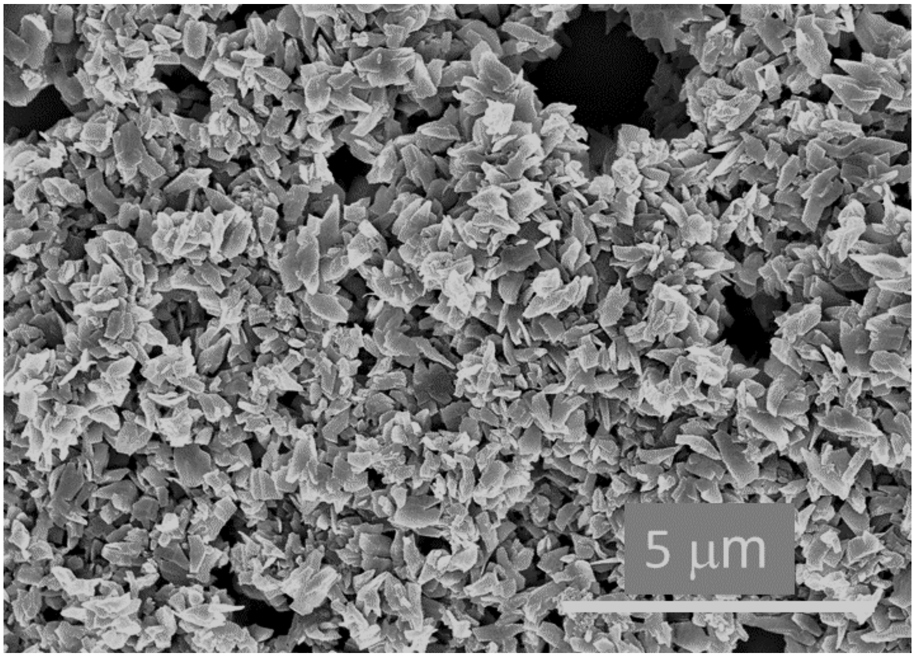

Figure S8: A low magnification image shown of crystals shown in Figure 6d, where crystals were produced under standard conditions with stirring.

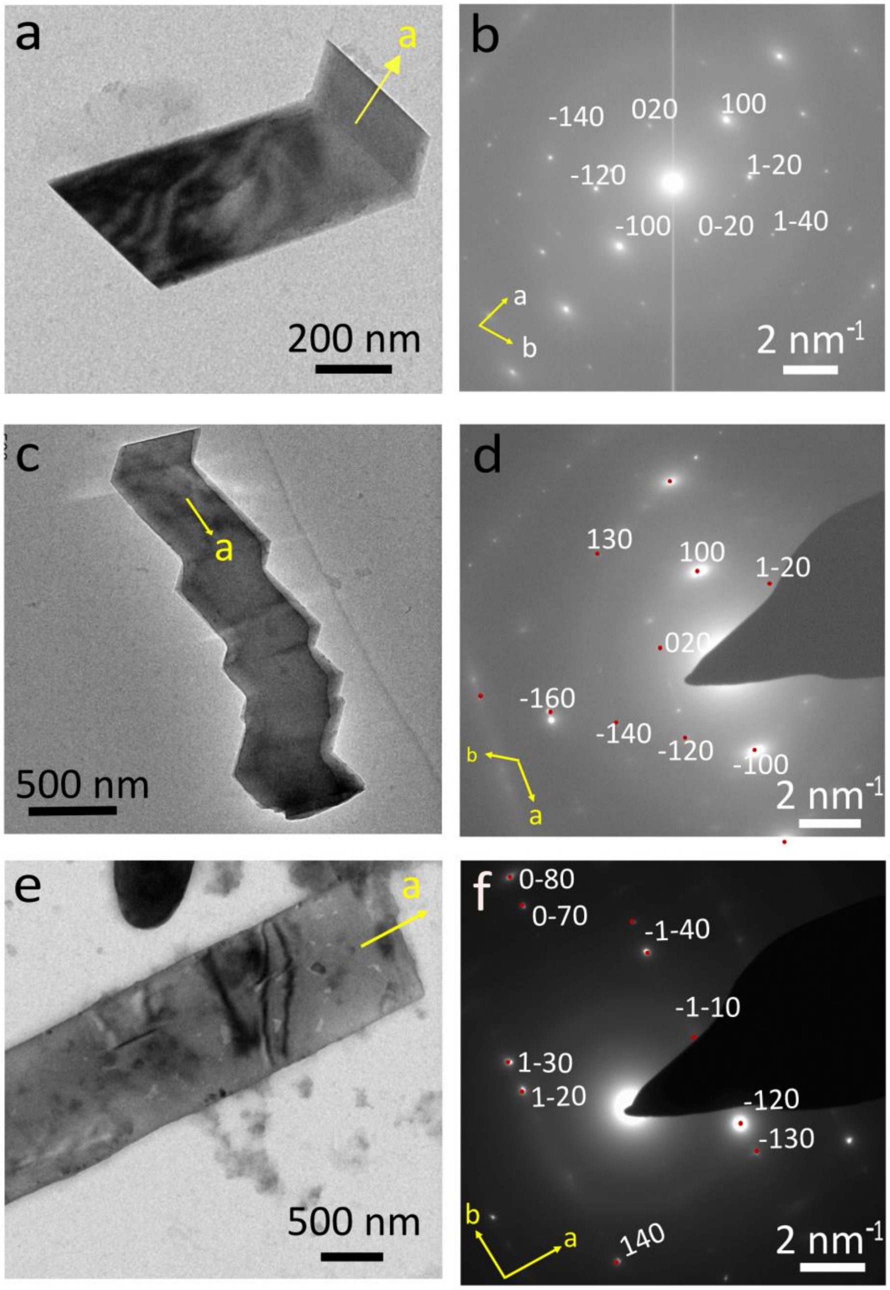

Figure S9: TEM images and the corresponding diffraction patterns of guanine crystals precipitated under standard conditions. (a,b) β-AG produced from the solution in the presence of orange G , (c,d) β-AG produced from the solution in the presence of hypoxanthine, and (e,f) α-AG produced from the solution in the presence of PVP.

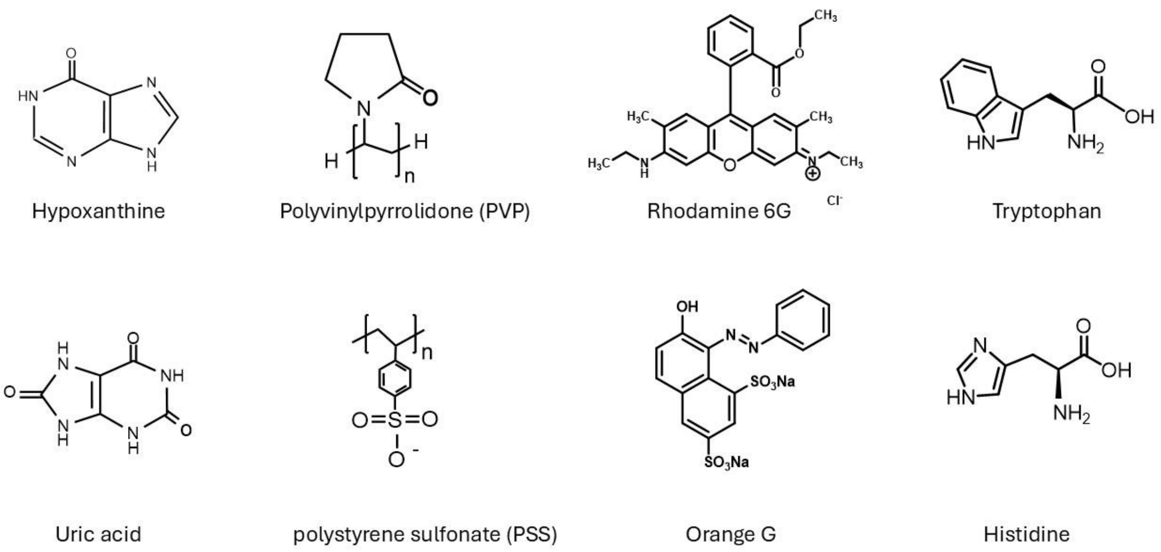

Figure S10: Chemical structures of the additives used in this work.

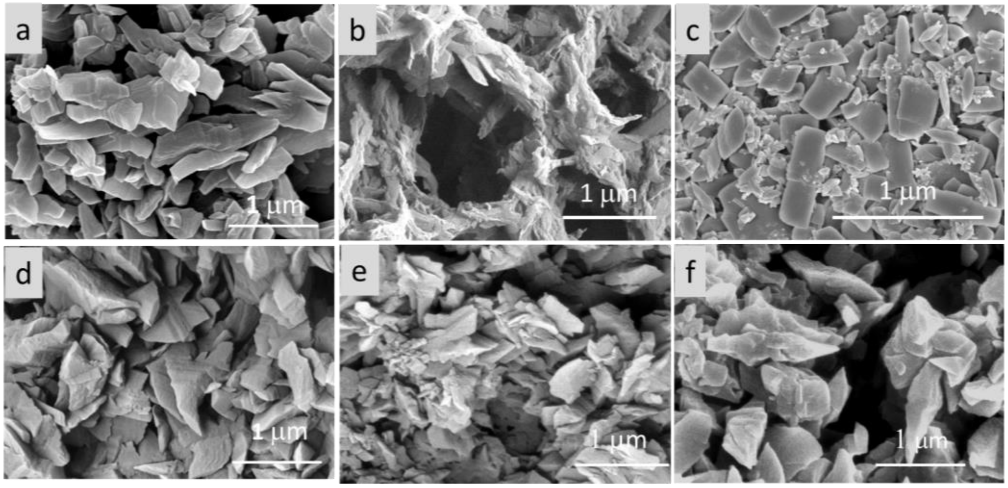

Figure S11. SEM images of crystals obtained after 1 day of reaction with 1 unit of PNP under stirring: (a) without additive, and with 0.5 mg mL^{-1} of (b) uric acid, (c) PVP, (d) orange G, (e) tryptophan, and (f) histidine.

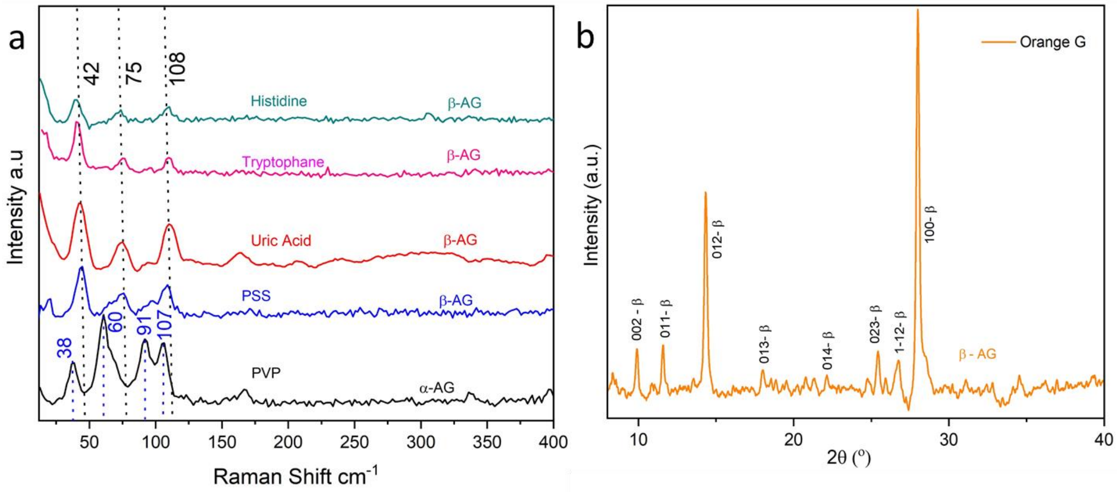

Figure S12: (a) Raman spectra of crystals formed after the addition of amino acids, purines, and polymer additives (0.5 mg mL^{-1}) with 1 unit of PNP, following 1 day of reaction under stirring. (b) PXRD patterns of crystals obtained after the addition of the dye orange G (0.5 mg mL^{-1}) with 1 unit of PNP, following 1 day of reaction under stirring.

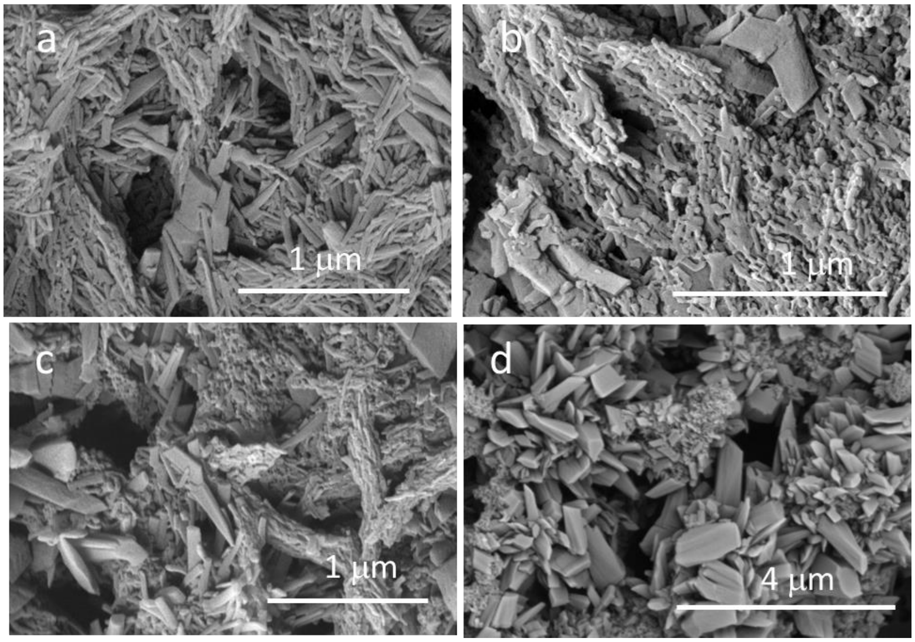

Figure S13. SEM images of crystals obtained at high pH with 1 unit of PNP with 0.5 mg mL^{-1} of (a) uric acid, (b) rhodamine 6G, (c) PVP, (d) tryptophan.

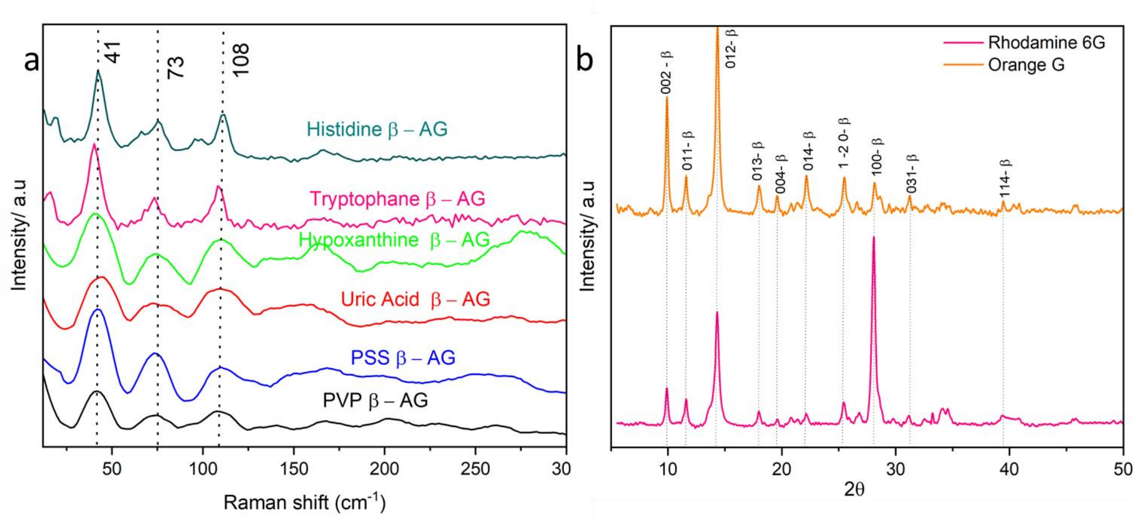

Figure S14: (a) Raman spectra of crystals formed at high pH after the addition of amino acids, purine, and polymer additives (0.5 mg mL^{-1}). (b) PXRD patterns of crystals obtained at high pH after the addition of dyes (rhodamine 6G, orange G, 0.5 mg mL^{-1}).

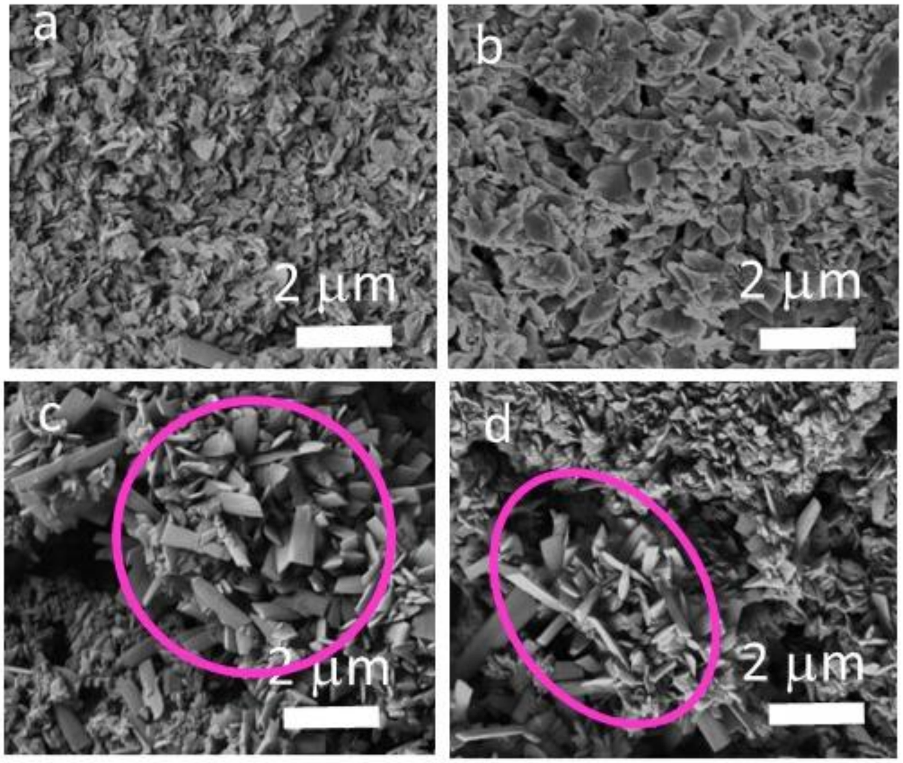

Figure S15. SEM images of crystals obtained after incubating β-AG crystals in (a) air, b) the mother solution, (c) DI water and (d) 50 mM HEPES buffer for 18 h. The pink circles highlight α-AG crystals with plate-like morphologies.

REFERENCE

1. Mao, C.; Cook, W. J.; Zhou, M.; Koszalka, G. W.; Krenitsky, T. A.; Ealick, S. E., The Crystal Structure of Escherichia Coli Purine Nucleoside Phosphorylase: a Comparison with the Human Enzyme Reveals a Conserved Topology. *Structure* **1997,** *5* (10), 1373-83.
